# Secular trends of endoscopic sleeve gastroplasty: analysis of the MBSAQIP database

**DOI:** 10.1007/s00464-026-13022-x

**Published:** 2026-07-07

**Authors:** Benjamin M. Moy, Rolando Barajas, Chloe M. Savino, Allison R. Schulman

**Affiliations:** 1 Department of Medicine, McGaw Medical Center of Northwestern University, Chicago, IL USA; 2 Division of Gastroenterology and Hepatology, University of Michigan, Taubman Center 3912, 1500 East Medical Center Drive, Ann Arbor, MI 48109 USA; 3 Department of Surgery, University of Michigan, Ann Arbor, MI USA

**Keywords:** ESG, Trends, Obesity, Adverse events, Incidence

## Abstract

**Background and aims:**

Endoscopic sleeve gastroplasty (ESG) is a minimally invasive endoluminal procedure for the management of obesity and has become increasingly popular in patients who are poor surgical candidates or who choose against surgical intervention. This study aims to evaluate secular trends of ESG in a large bariatric procedure-specific clinical database.

**Methods:**

We analyzed patients who underwent ESG from 2020 to 2023 from the Metabolic and Bariatric Surgery Accreditation and Quality Improvement Program (MBSAQIP). Annual incidence, patient characteristics and trends in 30-day serious adverse events following ESG were collected.

**Results:**

A total of 2371 patients (mean age 45y, 85.8% F, mean BMI 40 kg/m^2^) underwent ESG between 2020 and 2023. The number of ESGs increased by 50.5% across the study period. ESG volume increased significantly over time (IRR 1.11 per year, 95% CI 1.07–1.15; *p* < 0.01). Mean BMI increased 7.9% across the study period, rising from 37.8 kg/m^2^ to 40.8 kg/m^2^, respectively (*p* < 0.01) (Fig. 1B). Despite the rise in BMI and other pre-procedural comorbidities, there was no statistically significant difference in the incidence of adverse events across the study period.

**Conclusion:**

The number of ESGs being performed has significantly increased over the past few years. Despite expanding ESG to a higher-risk patient population, the incidence of serious adverse events following ESG has remained extremely low with no significant increase over the study period. As endoscopic techniques continue to evolve, ESG acts as a model for the future of endoluminal therapies in high-risk patient populations.

The obesity epidemic has continued to grow, affecting nearly 4 in 10 adults in the United States [1]. Traditionally, bariatric surgery has been the most effective treatment for obesity and obesity-related comorbidities, although fewer than 1–2% of eligible patients with obesity undergo bariatric surgery for a variety of reasons including side effects, surgical complications, and systemic barriers to access [2–4].

The field of endoscopic bariatric and metabolic therapy (EBMT) has gained widespread attention as a bridge between conservative measures such as lifestyle interventions or pharmacotherapy and more aggressive surgical interventions [2, 5, 6]. In particular, endoscopic sleeve gastroplasty (ESG) has been shown to be safe and effective for treatment of obesity, especially in patients who are poor surgical candidates, choose not to elect surgical intervention, or fail medical weight loss therapy. ESG is a gastric remodeling procedure that is performed using an endoscopic suturing device which facilitates imbrication of the greater curvature of the stomach and reduction of luminal volume by 70–80%.

With the growing adoption of ESG for the treatment of obesity, the Metabolic and Bariatric Surgery Accreditation and Quality Improvement Program (MBSAQIP) has been adapted to incorporate ESG in its data collection practices and promote standardization and quality improvement across accredited centers. Although procedural technique and patient care have evolved in recent years, little is known about the contemporary trends of ESG at the population level. This study aims to evaluate the incidence, patient characteristics, and 30-day post-procedural adverse events of ESG in a large bariatric procedure-specific clinical database.

## Methods

### Study population

This study retrospectively analyzed patients who underwent ESG or bariatric surgery from 2020 to 2023 from the Metabolic and Bariatric Surgery Accreditation and Quality Improvement Program  (MBSAQIP) Participant Use File (PUF), which captures all metabolic and bariatric surgical procedures and interventions entered into the MBSAQIP registry from over 950 centers in the United States and Canada. Case exclusion criteria include patients admitted to the hospital who have an included procedure to address cancer or trauma injury, patients under 5 years of age, patients with multiple assessed cases within 30 days, and cases completed due to complications or occurrences from a prior metabolic and bariatric procedure. To mitigate errors or discrepancies in data entry, all data are subject to an integrity audit to assess the quality of data, with a standardized data integrity audit disagreement rate of 5% across all participating centers. Hospital exclusion criteria include centers for whom the 30-day follow-up rate is < 80% or data integrity audit disagreement rate is > 5% and centers non-compliant with the MBSAQIP standards for data collection or annual metabolic and bariatric surgery clinical reviewer (MBSCR) certification exam. In compliance with privacy requirements of the American College of Surgeons, the MBSAQIP Participant Use File (PUF) contains only de-identified patient data and does not provide direct patient identifying information, protected health information, or specific hospital or provider data. Facility identifiers and geographic information regarding the accredited centers were also censored.

### Study variables

Demographic data from patients undergoing ESG were collected including sex, age, race, pre-procedural weight and body mass index (BMI), functional status, and smoking history. Patient comorbidities were also documented including a pre-procedural history of diabetes mellitus, chronic obstructive pulmonary disease (COPD), obstructive sleep apnea (OSA), pulmonary embolism (PE), gastroesophageal reflux disease (GERD), myocardial infarction, heart failure, hypertension, hyperlipidemia, deep vein thrombosis (DVT) or venous stasis, or renal insufficiency. Prior percutaneous coronary intervention or cardiac surgery, use of anticoagulation, presence of an inferior vena cava (IVC) filter, or use of dialysis were recorded.

The following 30-day post-procedural adverse events (AEs) and time to event were recorded: pneumonia, pulmonary embolism, ventilator dependence > 48 h, progressive renal insufficiency, acute renal failure, urinary tract infection, stroke or cerebrovascular accident (CVA), venous thrombosis requiring therapy, *Clostridium difficile* (*C. difficile*) colitis, sepsis, septic shock, gastrointestinal tract bleed, or bowel obstruction. Intra-procedural or post-procedural myocardial infarction (MI) or cardiac arrest requiring cardiopulmonary resuscitation (CPR) were also recorded.

### Outcomes

The primary outcome evaluated in this study included the annual incidence of ESG and patient characteristics across the study period. Secondary outcomes included the trends and frequency of 30-day post-procedural adverse events and the incidence of bariatric surgical procedures during this timeframe.

### Statistical analyses

Univariate descriptive statistics were summarized as frequencies (percentages) for categorical variables and as means with standard deviations or, when appropriate, medians with interquartile ranges for continuous variables. Bivariate comparisons were performed using chi-square or Fisher’s exact tests for categorical variables and analysis of variance (ANOVA) for continuous and ordinal variables. Poisson regression with year modeled as a continuous variable was performed for procedure count data. A two-tailed *P*-value of < 0.05 was considered statistically significant. All analyses were conducted using SAS version 9.4 (SAS Institute Inc., Cary, NC) and GraphPad Prism (Dotmatics, Boston, MA).

## Results

A total of 2371 patients were included in this study. Demographic information is shown in Table 1. The mean age of the cohort was 45 ± 10.7 years. The study comprised 85.8% (*n* = 2035) female and 14.2% (*n* = 336) male patients. The baseline mean BMI of the cohort was 40 ± 7.6 kg/m^2^.

**Table 1:** Demographic characteristics

Characteristic	Cohort (*n* = 2371)
Age	
Mean (SD)	45 (10.7)
Median (IQR)	44 (15)
Range	17–75
BMI (kg/m^2^)	
Mean (SD)	40 (7.8)
Median (IQR)	38 (9.9)
Range	16–92
Sex	
Female	2035 (85.8)
Male	336 (14.2)
Race	
American Indian or Alaska native	8 (0.3)
Asian	22 (0.9)
Black	382 (16.1)
Native Hawaiian or other Pacific Islander	4 (0.2)
White	1314 (55.4)
Mixed/Unknown	641 (27.1)

Categorical characteristics are presented as *n* (%)

**Fig. 1:**
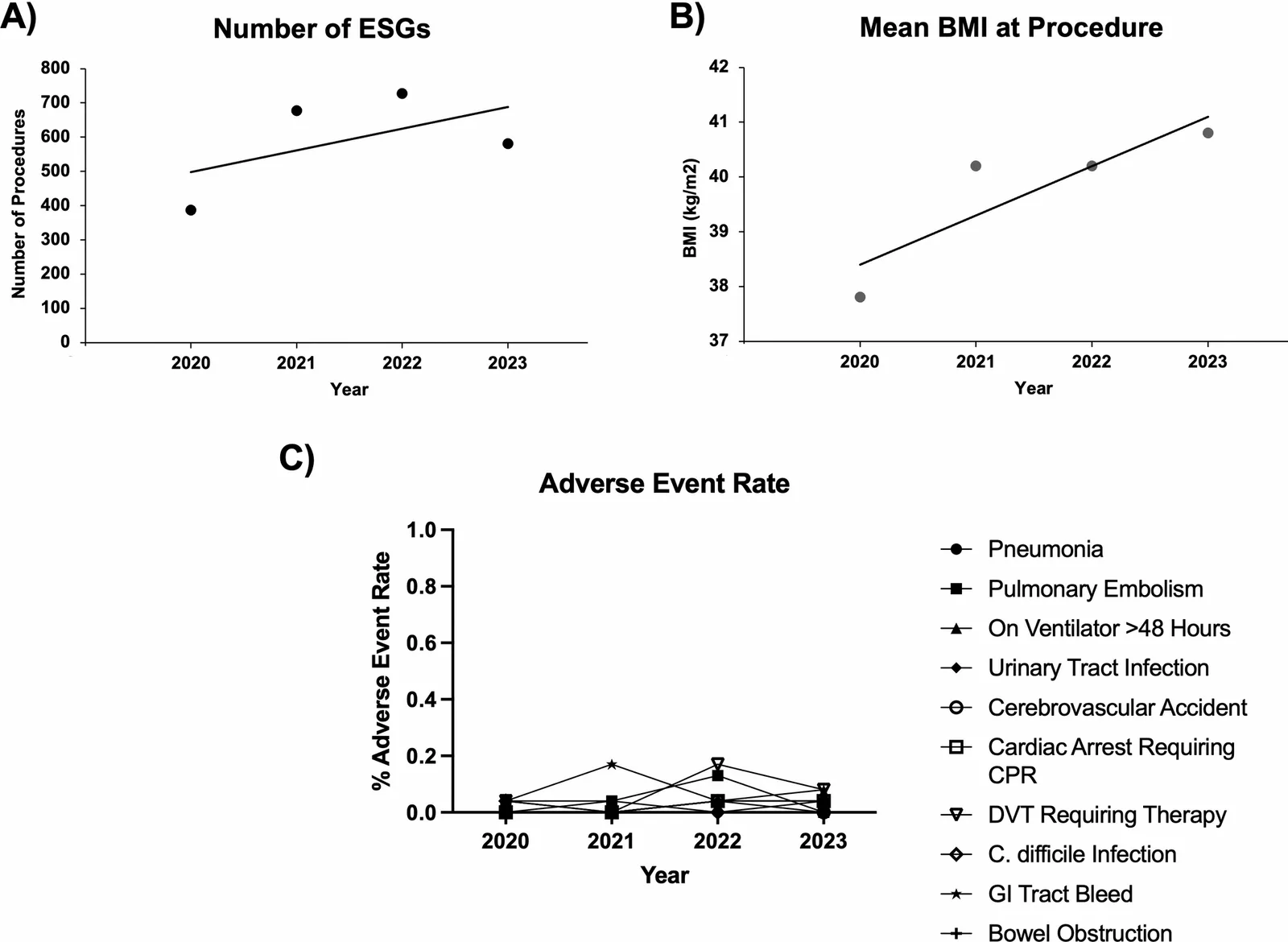
Procedural and demographic trends of ESG across study period

The number of ESGs increased by 50.5% between 2020 and 2023, rising from 386 to 581 (Fig. 1A). In a Poisson regression with year modeled as a continuous variable, ESG volume increased significantly over time (IRR 1.11 per year, 95% CI 1.07–1.15; *p* < 0.01). The incidence of sleeve gastrectomy and Roux-en-Y gastric bypass increased by 23.3% and 41.0%, respectively, during the study period.

The mean BMI in patients undergoing ESG increased 7.9% between 2020 and 2023, rising from 37.8 to 40.8 kg/m^2^, respectively (*p* < 0.01) (Fig. 1B**)**. There was also a significant increase in the number of patients with pre-procedural obstructive sleep apnea (*p* = 0.02) or anticoagulant use (*p* = 0.02). Despite the increase in BMI and other pre-procedural comorbidities over the study period, there was no statistically significant difference in the incidence of adverse events (AEs) across the study period including pneumonia (*p* = 0.55), pulmonary embolism (*p* = 0.93), ventilator requirement > 48 h (*p* = 0.11), urinary tract infection (*p* = 0.11), cerebrovascular accident (*p* = 0.74), cardiac arrest requiring CPR (*p* = 0.11), deep vein thrombosis requiring therapy (*p* = 0.49), *C. difficile* infection (*p* = 0.23), gastrointestinal tract bleed (*p* = 0.98), and bowel obstruction (*p* = 0.11) (Fig. 1C).

## Discussion

Despite the widespread adoption of ESG for the treatment of obesity, there is a paucity of literature on secular trends of ESG at the population level. As a result, little is known about the evolving demographics and risk profiles of patients undergoing these procedures and changes in 30-day adverse events on a large scale. This study is the largest to date characterizing contemporary trends of ESG using data from a nationwide bariatric consortium of accredited centers.

This study demonstrates an overall increase in the number of ESGs being performed, with an increase of 50.5% between 2020 and 2023 and a statistically significant average annual increase of 11% based on Poisson regression. This growth exceeds that of bariatric surgical procedures. Throughout the study period, most patients were functionally independent and had conditions associated with obesity including GERD, sleep apnea, hyperlipidemia, and diabetes. Despite these metabolic factors, ESG appears to be an increasingly appealing option for weight loss. Interestingly, the mean BMI increased nearly 8% across the study period, and an increasing number of patients with OSA and/or need for anticoagulation were electing to undergo these procedures.

Despite the expansion of this procedure to a higher risk patient population, the incidence of serious adverse events following ESG remained extremely low with no significant increase over the study period. This finding supports the safety of this procedure. The most common serious adverse events were gastrointestinal tract bleed, DVT, and PE, all of which occurred in < 0.5% of cases and within the first 14 days following ESG.

Prior research involving smaller cohorts confirms the findings of this study. The only randomized control trial involving patients undergoing ESG demonstrated a serious adverse event rate of only 2%, with no mortality or admissions to an intensive care unit [7]. Furthermore, the largest prospective case series of 1000 patients undergoing ESG demonstrated that fewer than 1% of patients were readmitted for fever, blood loss, or severe abdominal pain/nausea [8]. The current study further supports the safety of ESG as evidenced by low rates of post-procedural complications.

There are several limitations to our analysis. First, this data only encompasses ESGs being performed by proceduralists at MBSAQIP-accredited centers. As a result, there likely are differences in the practice patterns and experience of proceduralists. Furthermore, the lack of standardization and heterogeneity in ESG technique, in addition to individual center volume and learning curves, may play a significant role in the study outcomes. Notably, a portion of the patient population analyzed underwent procedures during the COVID pandemic. Challenges with staffing and the reduction in elective procedures during this time may have affected the number of ESGs performed. Additionally, all outcomes were reported at 30 days following ESG, therefore limiting this analysis to the immediate post-procedural period. Regardless, these data highlight the low adverse event rate of ESG within 30 days, a period during which patients are often at a heightened risk for post-procedural events. Moreover, large databases are subject to errors or discrepancies in data entry, and this may be particularly relevant for this study given variability and inconsistency in procedural coding for ESG across centers. However, all MBSAQIP data is subject to an integrity audit to assess the data quality, with a standardized audit disagreement rate of 5% across all participating centers. Centers for whom the Data Integrity Audit disagreement rate was > 5% were excluded.

In summary, there has been a steady adoption of reversible, minimally invasive endoluminal procedures for obesity such as ESG, which are becoming globally recognized for their favorable safety and efficacy profiles. The number of ESGs performed has increased since 2020, and patients undergoing these procedures appear to be at a higher risk with an increasing pre-procedural BMI and other obesity-related comorbidities. Despite these trends, the incidence of serious adverse events following ESG has remained extremely low with no significant increase over the study period. As the field continues to evolve with less invasive and more broadly adoptable techniques, ESG has set the standard for the growth of future endoluminal procedures in high-risk patient populations.
